# How does hepatic lipid accumulation lead to lipotoxicity in non-alcoholic fatty liver disease?

**DOI:** 10.1007/s12072-020-10121-2

**Published:** 2021-02-06

**Authors:** Yana Geng, Klaas Nico Faber, Vincent E. de Meijer, Hans Blokzijl, Han Moshage

**Affiliations:** 1 Department of Gastroenterology and Hepatology, University Medical Center Groningen, University of Groningen, Hanzeplein 1, 9713 GZ Groningen, The Netherlands; 2 Department of Laboratory Medicine, University Medical Center Groningen, University of Groningen, Groningen, The Netherlands; 3 Department of Hepatopancreatobiliary Surgery and Liver Transplantation, University of Groningen, University Medical Center Groningen, Groningen, The Netherlands

**Keywords:** NAFLD, NASH, MAFLD, Free fatty acids, Lipotoxicity, Lipid metabolism, Cell death, ER stress, Mitochondrial dysfunction, JNK

## Abstract

**Background:**

Non-alcoholic fatty liver disease (NAFLD), characterized as excess lipid accumulation in the liver which is not due to alcohol use, has emerged as one of the major health problems around the world. The dysregulated lipid metabolism creates a lipotoxic environment which promotes the development of NAFLD, especially the progression from simple steatosis (NAFL) to non-alcoholic steatohepatitis (NASH).

**Purposeand Aim:**

This review focuses on the mechanisms of lipid accumulation in the liver, with an emphasis on the metabolic fate of free fatty acids (FFAs) in NAFLD and presents an update on the relevant cellular processes/mechanisms that are involved in lipotoxicity. The changes in the levels of various lipid species that result from the imbalance between lipolysis/lipid uptake/lipogenesis and lipid oxidation/secretion can cause organellar dysfunction, e.g. ER stress, mitochondrial dysfunction, lysosomal dysfunction, JNK activation, secretion of extracellular vesicles (EVs) and aggravate (or be exacerbated by) hypoxia which ultimately lead to cell death. The aim of this review is to provide an overview of how abnormal lipid metabolism leads to lipotoxicity and the cellular mechanisms of lipotoxicity in the context of NAFLD.

## Introduction

In line with the global epidemic of obesity and type 2 diabetes mellitus (T2DM) the prevalence of non-alcoholic fatty liver disease (NAFLD) is increasing throughout the world. Recently, a new definition of NAFLD was suggested by a panel of international experts: Metabolic Associated Fatty Liver Disease (MAFLD), which contains “positive criteria” and is independent of alcohol use to diagnose the disease [1]. However, considering that the majority of studies referred to in this review were based on criteria of NAFLD, we decided to use the nomenclature of NAFLD in the current review. NAFLD is the most common liver disorder in Western countries with a prevalence of approximately 25% [2]. It is characterized by excessive hepatic lipid deposition (steatosis) in the absence of excessive alcohol use or alternative causes. The spectrum of NAFLD ranges from simple steatosis (NAFL) to non-alcoholic steatohepatitis (NASH), fibrosis and ultimately cirrhosis with its known complications, such as decompensation and/or hepatocellular carcinoma [3]. From 2003 to 2014 NASH was the most rapidly growing indication for liver transplantation in the United States [4]. In the near future, NASH will become the leading indication for liver transplantation [5].

NAFLD is recognized as the hepatic manifestation of the metabolic syndrome. Abundant data link NAFLD with abdominal obesity, T2DM, hypertension and atherogenic dyslipidemia which are risk factors for cardiovascular disease (CVD) [6, 7]. Mortality in patients with NAFLD is mainly attributed to increased risk of death due to CVD and liver-related death. Fibrosis stage predicted both overall and disease-specific mortality [8].

From a clinical point of view, the full understanding of the mechanisms underlying the development of NAFLD and NASH is of extreme importance to treat the hepatic and extrahepatic complications of NAFLD. It is now generally recognized that the development of NAFLD is a multifactorial process. Apart from obesity and T2DM, which are two important factors commonly associated with NAFLD, environmental and genetic factors, as well as gut microbiota, are likely to play a role in the onset and progression of NAFLD [3, 9, 10]. A thorough discussion of the role of microbiota and NAFLD is outside the scope of this review and has been reviewed recently [11]. Thus, both the pathophysiological mechanisms leading to disease and the clinical manifestations are highly heterogeneous. Hepatic steatosis results from an imbalance between synthesis and utilization of lipids. Dysregulation of lipid homeostasis in hepatocytes leads to the generation of toxic lipids that result in dysfunctional organelles promoting inflammation, hepatocellular damage and cell demise [8]. Elucidating the pathways leading to lipotoxicity-induced cell injury may lead to the development of new therapeutic options. Thus, the various pathways involved in hepatic fat accumulation and lipid toxicity contributing to the pathogenesis of NAFLD and eventually end-stage liver disease are discussed below.

## Metabolic changes of lipids in non-alcoholic fatty liver disease

### Lipolysis of adipose tissue

Most NAFLD patients have increased white adipose tissue (WAT) mass and its expansion is a high-risk factor in the progression of NAFLD [12, 13]. Human studies have shown that the shift of fat storage from subcutaneous to visceral adipose tissue is associated with liver damage [12, 14] and the expansion of visceral adipose tissue may be a powerful predictor for NAFLD. Nonetheless, determining whether the liver damage is associated with the changes in the fatty acid composition of adipose tissues needs more study [15].

As the main source of circulating lipids, the increased secretion of FFAs from adipose tissue is strongly associated with enhanced lipolysis, in particular increased triglyceride (TG) hydrolysis. Obese NAFLD patients demonstrated greater lipolysis in adipose tissue, which may account for 60–70% of fat accumulating in the liver [16]. The excessive lipolysis of adipose tissue is accompanied with abnormal production of hormones released by adipocytes (e.g. reduced production of adiponectin) and adipose tissue inflammation (e.g. the release of pro-inflammatory cytokines), all of which participate in promoting insulin resistance (IR), thus further contributing to ectopic fat deposition [17, 18].

**Fig. 1:**
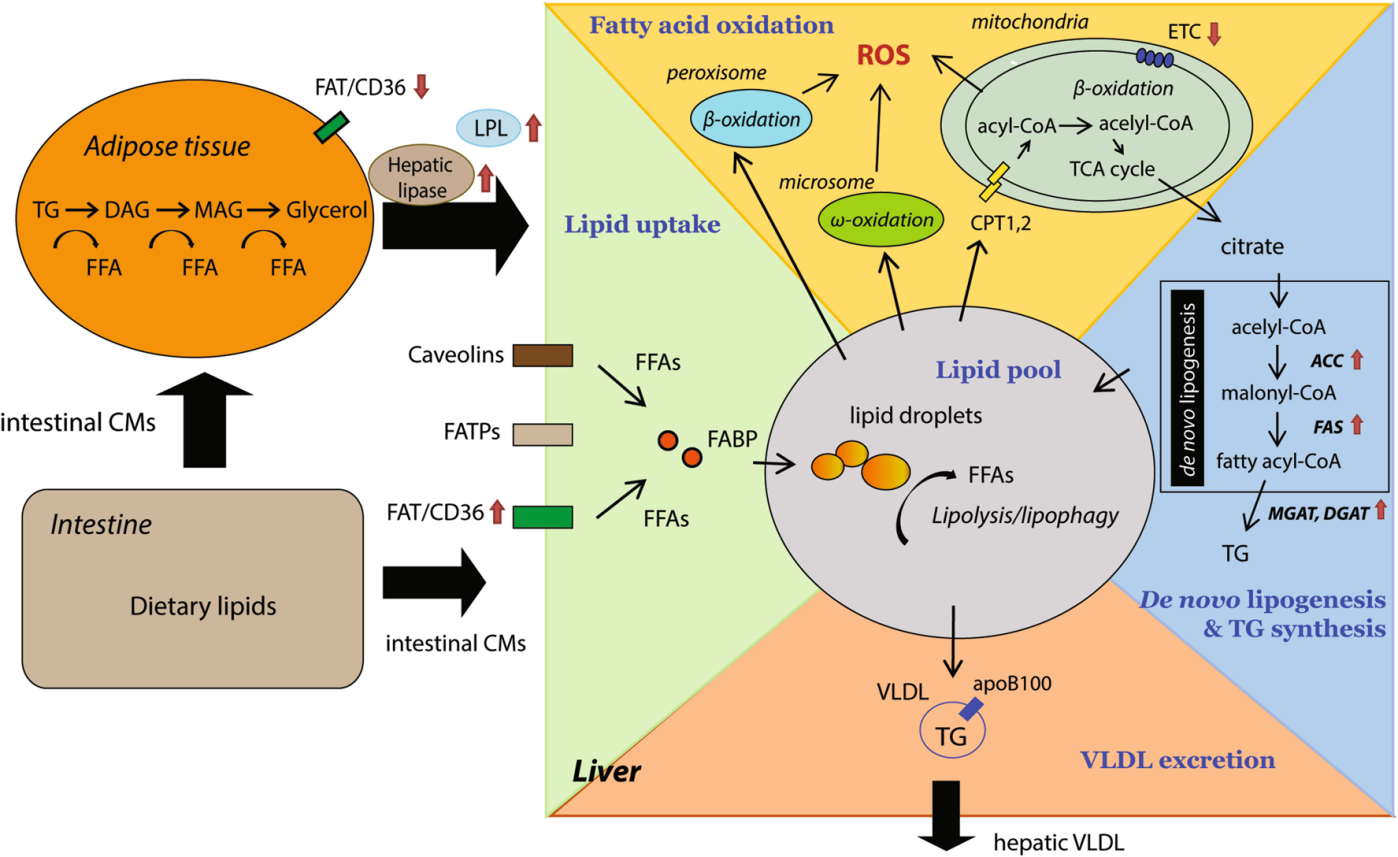
Hepatic lipid metabolism in NAFLD. Dietary lipids are mainly absorbed in the intestine and incorporated into intestinal chylomicrons (intestinal CMs) and then transported to adipose tissue and the liver via the bloodstream. In adipose tissue, intestinal CMs are taken up and stored in lipid droplet. In NAFLD, the hydrolysis of TG is largely enhanced in adipocytes. TG is hydrolyzed into diacylglycerol (DAG), monoacylglycerol (MAG) and glycerol, releasing one fatty acid in each step. The increased fatty acid load is then delivered to the liver, scavenged by fatty acid translocase (FAT/CD36), fatty acid transport proteins (FATPs) and caveolins and incorporated into lipid droplets. In hepatocytes, de novo lipogenesis is increased which contributes to the lipid pool as well. On the other hand, fatty acids are oxidized in mitochondria (mitochondrial β-oxidation), peroxisomes (peroxisomal β-oxidation) and microsomes (microsomal ω-oxidation), accompanied by an elevated production of ROS. The secretion of TG-enriched very-low-density lipoprotein (VLDL) is another route of lipid disposal. It is increased in absolute terms, but its increase does not match the increased fat accumulation in NAFLD. Arrows (red) indicate the up- or down-expressions of specific proteins in NAFLD. *CPT1/2* carnitine palmitoyltransferase ½, *FFA* free fatty acid, *TCA* cycle: the citric acid cycle, *ETC* electron transport chain, *FABP* fatty acid-binding protein, *ACC* acetyl-CoA carboxylase, *FAS* fatty acid synthase, *DGAT* diacylglycerol acyltransferase, *MGAT* monoacylglycerol acyltransferase

In addition to the cytosolic lipolysis, lipophagy is an alternative way of TG hydrolysis. As a form of autophagy, specifically macroautophagy, lipophagy mediates the hydrolysis of TG stored in lipid droplets via fusion of the engulfed droplets with lysosomes. Of note, the contribution of lipophagy to the catabolism of TG is more prominent in brown adipose tissue and the liver compared to WAT [19]. Moreover, NAFLD patients, subjected to chronic lipid exposure, exhibit an inhibited lipophagy, whereas an acute addition of lipids to cells in vitro may enhance lipophagy, which might be an adaptive response. In addition, FFAs derived from the efflux from other cell types, for example, hepatocytes, as well as FFAs released from TG-rich lipoproteins by lipoprotein lipase (LPL), are also sources of circulating FFAs (Fig. 1).

### Lipid uptake

In NAFLD patients, the uptake of circulating lipids, in particular FFAs and lipoproteins, by the liver is increased. This increased uptake is related to the enhanced level of lipid transport proteins in the plasma membrane, as well as increased levels of hepatic lipase and lipoprotein lipase (LPL) in response to IR [20, 21].

Fatty acid transport proteins (FATPs), caveolins and fatty acid translocase (FAT/CD36) are the main hepatic plasma membrane proteins that act as scavengers of lipids and contribute to the hepatic lipid pool. Among the FATP members, FATP2 and FATP5 are the main isoforms expressed in the liver. Silencing FATP2 or knockout of FATP5 led to reduced hepatic TG levels and resistance to diet-induced obesity in mice [9, 22]. On the other hand, NAFLD is associated with increased expression and translocation of caveolin-1, a protein functioning in cellular cholesterol transport [23]. Interestingly, caveolin-1 overexpression protected and caveolin-1 knockout aggravated hepatic steatosis in HFD-fed mice by affecting lipogenesis, which indicates regulatory functions of caveolin-1 other than lipid transport in the liver [24]. In NAFLD patients, the expression of FAT/CD36 is increased in the liver and correlated with hepatic apoptosis [9]. Moreover, hepatocyte-specific deletion of FAT/CD36 prevented fat accumulation in the liver and repressed inflammation [25]. Notably, in the liver, FAT/CD36 is highly expressed in non-parenchymal cells in comparison to hepatocytes, thus FAT/CD36 might play more important roles in hepatic inflammation and fibrogenesis compared to lipid uptake. With regards to the role of lipases, loss-of-function studies on hepatic lipase and LPL in the liver showed that knockout of hepatic lipase resulted in increased hepatic steatosis and inflammation in mice fed a high-fat high-cholesterol diet that might be due to the activation of stress pathways like SAPK/JNK and p38 [26]. LPL is mainly expressed in non-parenchymal cells in the liver and is also involved in hepatic fibrogenesis [27].

In addition, hepatic FABPs facilitate the shuttling of fatty acids and their expression positively correlates with the progression of NAFLD. Different FABPs transfer fatty acids to phospholipid bilayers at different rates and via distinct kinetic mechanisms [28]. Furthermore, FABPs may also participate in other cellular processes, such as cell differentiation [28]. Thus, more studies are needed to elucidate their role in the progression of NAFLD.

### De novo lipogenesis (DNL) and triglyceride synthesis

DNL is a cellular process that converts acetyl-CoA and malonyl-CoA into fatty acids. Several clinical studies indicated that the rate of DNL is increased and responsible for around 20–30% of fat accumulation in the liver in obese NAFLD patients [13, 16]. Mechanistically, the elevated DNL is closely associated with IR and excessive carbohydrate intake. IR hampers glucose oxidation and shuttles carbohydrates into the DNL pathway [20]. Of note, compared to glucose, fructose demonstrates a greater influence on activating DNL and fueling TG synthesis [29]. The elevated DNL is concomitant with increased de novo glycerolipid synthesis and subsequent TG accumulation, incorporating FFAs into TG and forming lipid droplets. Apart from facilitating TG synthesis, glycerolipid metabolism is a major process that produces a large number of lipid second messengers, including phosphatidic acid, lysophosphatidic acid, diacylglycerol (DAG), other lipid intermediates and related lipids (e.g. ceramides and various sphingolipids). These lipids have been linked to impaired insulin signaling and cytotoxic effects [30]. Although several lipidomics studies have shown that NAFLD patients demonstrated abnormal glycerolipid metabolism (summarized in 3.1), the underlying molecular mechanisms are still largely unclear.

### Fatty acid oxidation (FAO)

Fatty acid oxidation (FAO) is roughly proportional to the concentration of plasma FFAs. Within cells, FFAs, either imported from plasma or released from intracellular lipids via cytosolic lipolysis or lipophagy, are mainly oxidized in mitochondria (mitochondrial β-oxidation), peroxisomes (peroxisomal β-oxidation) and microsomes (microsomal ω-oxidation). Although mitochondrial β-oxidation is one of the best-studied processes, contradictory results were reported in subjects with NAFLD. Since there is no direct measurement of mitochondrial oxidation available in vivo, indirect measures assessing plasma ketone body concentrations suggest that hepatic FAO could be either increased or decreased [13, 31]. Similarly, animal and human studies using stable isotopes showed increased, normal or decreased FAO [32–34]. Notably, the activities of all mitochondrial respiratory chain complexes were undisputedly decreased in the liver tissue of patients with NASH [34].

Besides mitochondria, fatty acids can also be oxidized in peroxisomes and microsomes. These two organelles are mainly responsible for the oxidation of very-long-chain fatty acids, branched-chain fatty acids and unsaturated fatty acids. Deficiency of peroxisomal enzymes [e.g. acyl-coenzyme A oxidase (Acox1) or peroxisomal biogenesis factor 11α (Pex11α)] resulted in impaired peroxisomal β-oxidation, increased lipid accumulation and exacerbated hepatocellular damage [35, 36], indicating the indispensable role of peroxisomes in lipid metabolism in the context of NAFLD. With respect to microsomal ω-oxidation, hepatic fatty acid overload acts both as a substrate and as an inducer of microsomal cytochrome P-450 family members. Together with mitochondria, peroxisomes and microsomes are important sources of ROS production.

### Very low-density lipoprotein (VLDL) excretion

Hepatic TG-enriched lipoprotein secretion is increased in absolute terms in NAFLD, but its increase does not match the increased fat accumulation in hepatocytes [13, 37]. Moreover, NASH patients demonstrated impaired synthesis and excretion of lipoproteins [13, 38], indicating that there is a limited capability of excretion of VLDL particles, which might be secondary to hepatocellular impairment [38, 39]. In addition, NAFLD patients showed increased production of small dense LDL, which is a risk factor of CVD [40].

VLDL is structured as a TG-enriched core with a monolayer of phospholipids and incorporating proteins like apolipoprotein B-100 facilitating VLDL delivery and uptake. Interestingly, it has been shown that the bulk of TG incorporated into lipoprotein VLDL is derived from exogenous lipids, but not from DNL [16, 37]. The impaired apolipoprotein B-100 synthesis in NASH patients could be related to the increased cellular FFA level, disturbed redox balance, hyperinsulinemia and decreased expression, all of which were shown to hamper apolipoprotein B-100 synthesis and VLDL assembly, thus resulting in lipid accumulation in the liver [39].

## Mechanisms of lipotoxicity

**Fig. 2:**
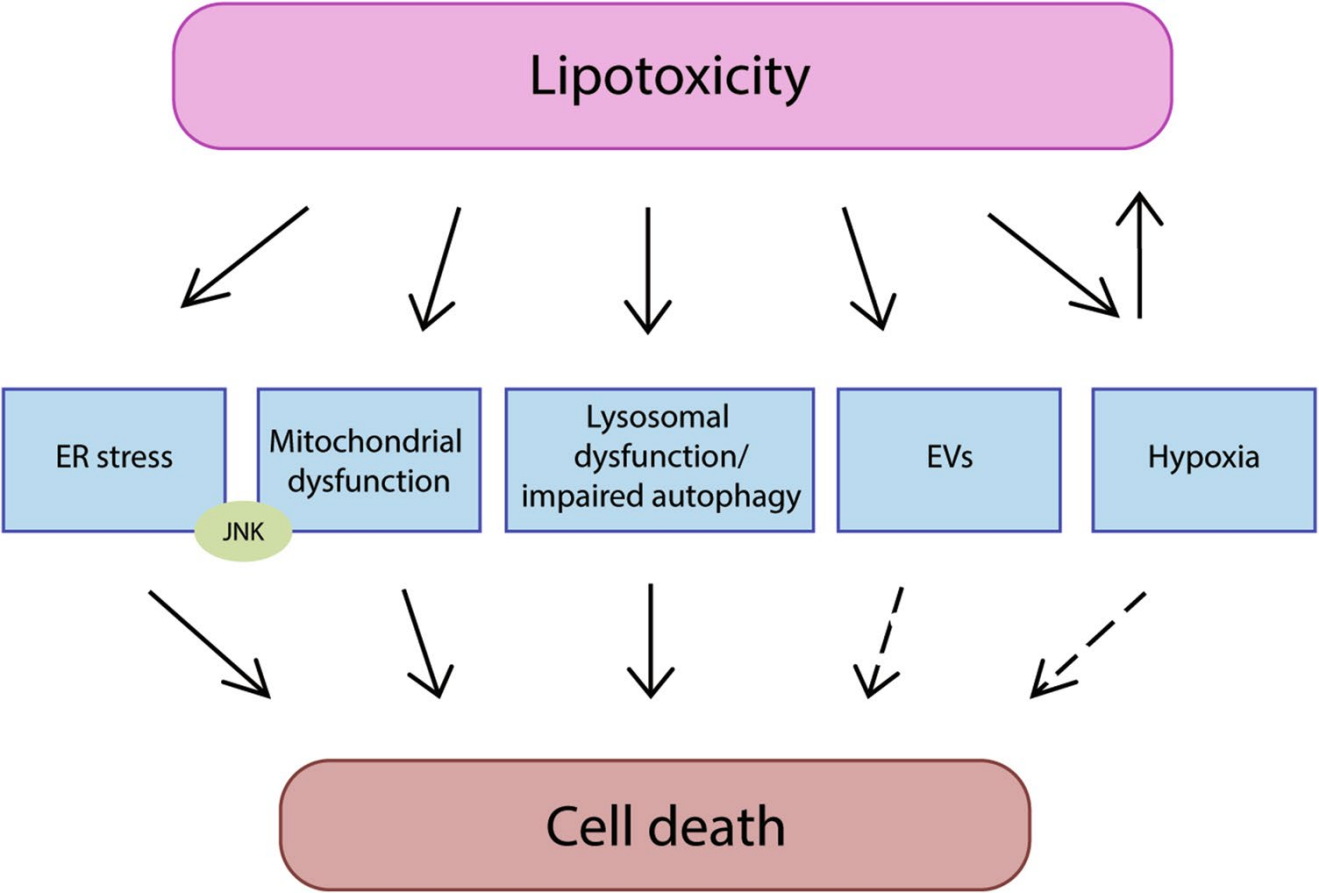
Mechanisms of lipotoxicity in NAFLD. The mechanisms of lipotoxicity involve several cellular processes, such as ER stress, mitochondrial dysfunction, lysosomal dysfunction, the release of extracellular vesicles (EVs) and hypoxia, ultimately leading to cell death. Additionally, the activation of JNK signaling pathway also plays an important role in lipoapoptosis and its activation is closely associated with ER stress/UPR and mitochondrial dysfunction

Lipotoxicity, defined as the toxicity of lipids leading to organellar dysfunction, abnormal activation of intracellular signaling pathways, chronic inflammation and cell demise, is a hallmark of NASH [30, 41, 42]. The mechanisms of lipotoxicity involve several cellular processes, such as ER stress, mitochondrial dysfunction and lysosomal permeabilization, ultimately leading to cell death (apoptosis, necroptosis or pyroptosis). In recent years it has been shown that lipotoxicity may induce the release of extracellular vesicles (EVs) and affects (and is affected by) hypoxia, both of which play important roles in the progression of NAFLD (Fig. 2). The following paragraphs provide an update of the recent findings on the mechanisms of lipotoxicity in NAFLD.

### Various lipid species are involved in non-alcoholic fatty liver disease

One of the diagnostic criteria of NAFLD is “more than 5% (v/v) fat accumulation in the liver” on histology. However, not all lipids are toxic, e.g. TGs, which are considered as an inert lipid species, are recognized to be protective against toxic lipids. Moreover, several mono- or poly-unsaturated fatty acids [MUFAs and PUFAs, e.g. oleic acid, docosahexaenoic acid (DHA) and eicosapentaenoic acid (EPA)] demonstrate beneficial actions in NAFLD by inhibiting oxidative stress and inflammation and reducing lipid deposition [30]. Meanwhile, the toxic lipids, such as saturated fatty acids (SFAs), free cholesterol (FC), glycerophospholipids (GPLs) and sphingolipids, cause cellular injury and may promote cell death [30].

Our knowledge about the lipid species involved in NAFLD has substantially increased in the last decade. The unique lipid signatures across the entire disease spectrum have been revealed in NAFLD patients owing to the progression in lipidomics. The summary of the main lipidomics studies from liver biopsies and plasma samples of NAFLD patients are presented in Tables 1 and 2.

**Table 1:** Lipidomics data from liver biopsies of patients

References	Year	Study cohort	Technique	Main findings
[43]	2007	Normal (*n* = 9) NAFL (*n* = 9) NASH (*n* = 9)	TLC	1. Increased TG and DG, but unaltered FFAs in both NAFL and NASH cohorts compared with control 2. FC and TG/DG ratio progressively increased from normal liver to NAFL to NASH. However, the total CE content was not significantly changed in either NAFL or NASH 3. n-6 and n-3 PUFAs within hepatic FFAs exhibited trends of stepwise decrease from control to NAFL to NASH 4. PUFAs in DG and TG decreased significantly, meanwhile, SFAs and MUFAs in DG and TG showed trends for increase in NAFL and NASH 5. The total PC decreased in both NAFL and NASH, but no significant changes in PS or CL 6. AA was relatively depleted from most lipid species
[44]	2015	Normal (*n* = 31) Steatosis (*n* = 17) NASH (*n* = 20) Cirrhosis (*n* = 20)	LC–MS	1. TG was higher in NASH and steatosis cohorts compared with normal 2. TG contained higher amounts of short-chain FA- and SFA, lower amounts of PUFA in NASH compared with steatosis 3. PUFA-containing LyPEs were significantly increased in steatosis, but not in NASH 4. Almost all lipids except for eicosanoids and certain GPLs (e.g. PIs, ether-linked PEs) were lower in the cirrhotic than in normal samples 5. NASH demonstrated higher amounts of many sphingolipids and the greatest distinction between NASH and steatosis was in very-long-chain dihydroceramides and 1-deoxydihydroceramides
[45]	2017	Control (*n* = 7) NAFL (*n* = 39) NASH (*n* = 15)	GC/LC–MS	1. The increased FAs in NASH (14:0, 16:0, 16:1 n-7, 18:1 n-7, 18:1 n-9 and 18:2 n-6) belong to the long-chain FA synthesis pathway and related to the decreased ELOVL6 activity and increased FADS2 and SCD1 activities 2. The significantly decreased eicosanoid precursors (AA, EPA and DHA) is related to the decrease in FADS1 activity in NASH compared with control 3. The deficiency in the synthesis of polyunsaturated long-chain FA causes a decrease in phospholipids in NASH patients
[46] Focused on mitochondrial lipids	2018	Control (*n* = 16) NAFL (*n* = 10) NASH (*n* = 32)	LC–MS	1. The levels of TG, DG, dihydroceramide, cholesterol, CE, acylcarnitine, dihexosylceramide, CL and ubiquinone were significantly higher in NAFL and/or NASH compared with control 2. SFAs (e.g., 14:0, 17:0, and 18:0) in TG increased significantly in NASH compared with control 3. Only acylcarnitine and dihexosylceramide demonstrated a significant increase in NASH compared with NAFL 4. Hepatic CL and ubiquinone accumulated in NAFL and NASH, and levels of acylcarnitine increased in NASH, indicating the mitochondrial dysfunction. However, the percentage of 18:2 FA in CL, the most abundant FA composition in CL, was lower in NASH 5. Among the most abundant FAs in CEs, 16:1 and 18:2 FAs were increased, while 16:0 and 18:1 FAs were decreased in NASH compared with control. Meanwhile, 16:0 FA in CEs was also significantly lower in NAFL compared with control 6. The percentage of polyunsaturated AA in TG showed a stepwise decrease from Control to NAFL to NASH 7. Only dihydroceramide and dihexosylceramide were significantly different (increased) in the sphingolipids class between the control and NAFL/NASH 8. NAFL and NASH demonstrated higher amounts of MUFAs (16:1 and 18:1), however lower amounts of SFA (18:0), diunsaturated (DUFA, 18:2), and long-chain PUFAs (20:4, 22:5, and 22:6) in DGs 9. Several phospholipid species comprising DHA decreased during the progression of NAFLD, including PC(P-16:0/22:6), LyPC (22:6), and PE(18:1, 22:6)

*NAFL* non-alcoholic fatty liver, *TLC* thin-layer chromatography, *FC* free cholesterol, *CE* cholesterol ester, *PC* phosphatidylcholine, *CL* cardiolipin, *PUFAs* polyunsaturated fatty acids, *SFAs* saturated fatty acids, *MUFAs* monounsaturated fatty acids, *AA* arachidonic acid, *DHA* docosahexaenoic acid (22:6 n-3), *EPA* eicosapentaenoic acid (20:5 n-3), *PE* phosphatidylethanolamine, *LyPC* lysophosphatidylcholine, *PS* phosphatidylserine, *SM* sphingomyelin, *LyPE* lysophosphatidylethanolamine, *GPL* glycerophospholipid, PI phosphatidylinositol, *FA* fatty acid, *ELOVL6* elongation of very long chain fatty acids protein 6, *FADS* fatty acid desaturase, *SCD1* stearoyl-CoA desaturase 1

**Table 2:** Lipidomics data from plasma samples of patients

References	Year	Study cohort	Technique	Main findings
[47]	2009	Normal (*n* = 50) NAFL (*n* = 25) NASH (*n* = 50)	TLC	1. The total plasma MUFAs, which were driven by palmitoleic (16:1) and oleic (18:1) acids, were significantly increased in NAFL and NASH 2. The total SFAs were higher in both NAFL and NASH compared with control 3. The levels of DG and TG were significantly lower in NAFL and NASH compared with control 4. The total n-3 and n-6 PUFAs content progressively decreased in most lipid classes (including FFAs, TG, PC, LyPC) 5. Linoleic acid (8:2) was decreased with a concomitant increase in γ-linolenic (18:3) and dihomo γ-linolenic (20:3) acids in both NAFL and NASH 6. The plasma levels of AA (prostaglandin [PG]-M, PGB2, PGD2, PGE2, PGF2, and thromboxane A2) were not significantly different among normal, NAFL and NASH 7. The plasma levels of several products of the LOX pathway (5-HETE, 8-HETE, and 15-HETE) were significantly higher in NASH compared with NAFL/normal
[44]	2015	Normal (*n* = 31) Steatosis (*n* = 17) NASH (*n* = 20) Cirrhosis (*n* = 20)	LC–MS	1. The plasma levels of several eicosanoids and FFAs were increased, whereas the plasma levels of many plasma neutral lipids, GPLs, and some sterols were reduced in cirrhosis, but not in NASH 2. Cirrhotic patients demonstrated decreased plasma levels of most SMs, long-chain bases, and glycolytic/TCA cycle metabolites, while increased plasma levels of several glucosylceramide species and nucleotides 3. NASH patients exhibited elevated ceramides, dihydroceramides, and 1-deoxy-dihydroceramides and 1-deoxy-ceramides 4. CEs, some sphingolipids and GPLs (e.g. PUFA PEs and those containing ether-linked moieties were increased), and the aqueous metabolite F16BP could distinguish NASH from steatosis 5. Sphingolipids and GPLs, which provide the best clustering by linear discriminant analysis, could distinguish all the histological states
[48]	2016	Non-NAFLD (*n* = 132) NAFL (*n* = 117) NASH (*n* = 69)	UPLC-MS	1. NASH was significantly associated with the elevated concentrations of saturated and monounsaturated TGs in the serum 2. NASH exhibited significantly reduced levels of SMs and lyPC compared with other groups 3. The absolute concentrations of saturated and monounsaturated TGs were significantly increased in NASH compared with other groups 4. The levels of saturated and monounsaturated TGs were elevated in NASH compared with non-NAFLD, and was inversely correlated with the number of double bonds
[49] Focused on circulating phospholipids	2018	Normal (*n* = 28) NAFL (*n* = 25) NASH (*n* = 42)	LC–MS	1. Increased circulating PC and SM, while decreased PE in NAFL and NASH 2. NASH patients demonstrated elevated PE compared with NAFL subjects 3. LyPE was significantly decreased in NAFL and NASH compared with control 4. The levels of PI and plasmalogens were not significantly different among all groups 5. NAFLD patients with hypertension demonstrated higher amounts of circulating PC and SM than NAFLD patients without hypertension 6. Diabetic (*n* = 18) and non-diabetic NAFLD patients (*n* = 49) showed no difference in the total phospholipids, including PC, PE and SM
[46] Focused on mitochondr-ial lipids	2018	Control (*n* = 16) NAFL (*n* = 10) NASH (*n* = 32)	LC–MS	Acylcarnitines were increased in NASH compared with control

*NAFL* non-alcoholic fatty liver, *TLC* thin-layer chromatography, *FC* free cholesterol, *CE* cholesterol ester, *PC* phosphatidylcholine, *CL* cardiolipin, *PUFAs* SFAs: saturated fatty acids, *MUFAs* monounsaturated fatty acids, *AA* arachidonic acid, *DHA* docosahexanoic acid (22:6 n-3), *DPA* docosapentenoic acid (22:5 n-3), *PE* phosphatidylethanolamine, *LyPC* lysophosphatidylcholine, *COX* cyclooxygenase, *PS* phosphatidylserine, *SM* sphingomyelin, *TCA cycle* the citric acid cycle, *LOX* lipoxygenases, *HETE* hydroxyeicosatetraenoic acid, *LyPE* lysophosphatidylethanolamine, *GPL* glycerophospholipid, *PI* phosphatidylinositol, *F16BP* fructose-1,6-bisphosphate

Generally speaking, in the liver, there are increased levels of TG, diacylglycerol (DG) and FC in both NAFL and NASH patients, without substantial changes in the total FFA pool [43–46]. NASH patients showed higher amounts of short-chain FAs-, SFAs- and MUFAs-containing TG and lower amounts of PUFA-containing TG compared to control or NAFL subjects [43, 44, 46]. NASH was also associated with higher levels of sphingolipids and significantly decreased levels of arachidonic acid (AA) and AA-containing lipids [45, 46]. Regarding mitochondrial lipids, hepatic cardiolipin (CL) and ubiquinone are accumulated in NAFL and NASH, indicating increased mitochondrial oxidation and mitochondrial dysfunction. However, Puri et al. showed unaltered CL levels in the liver [43]. Furthermore, in cirrhotic patients, almost all lipids except eicosanoids and certain GPLs [e.g. phosphatidylinositols (PIs), ether-linked phosphatidylethanolamines (PEs)] were reduced compared to normal subjects [44].

Plasma from NAFL and NASH patients demonstrated a significant increase of TG, DG, MUFAs and SFAs, whereas a gradual decrease of total omega-3 and omega-6 PUFAs content was observed across most lipid classes [FFAs, TG, phosphatidylcholine (PC) and lysophosphatidylcholine (LyPC)] [47, 48]. In these studies, contradictory results were observed with regard to several classes of phospholipids. E.g. sphingomyelin (SM) and PC/LyPC were reported to be either increased [49] or decreased [48] in NASH. Furthermore, cirrhotic patients demonstrated reduced plasma neutral lipids, GPLs and some sterols, but no reduction of FAs and several eicosanoids [44].

### Endoplasmic reticulum (ER) stress

As the main site of both lipid synthesis and protein folding and maturation, the ER is an important participant in the development of NAFLD. In the lipotoxic environment of NAFLD, many factors were shown to cause ER stress, including disrupted ER-to-Golgi protein trafficking, altered ER membrane composition and stiffness, impaired VLDL-TG assembly and the activation of intracellular signaling pathways [50]. Moreover, obese individuals demonstrate a decreased level of sarco/endoplasmic reticulum Ca^2+^-ATPase 2 (SERCA2) activity, which pumps the cytosolic Ca^2+^ into the ER lumen [51]. The change of Ca^2+^ gradient is another factor that is implicated in the occurrence of ER stress. ER stress triggers the activation of the UPR, which is initially destined to attenuate cellular stress. However, long-term non-attenuated ER stress promotes cellular injury via the apoptotic signaling pathways of the UPR.

In mammalian cells, the UPR is a highly conserved cellular signaling response, which is composed of three ER transmembrane proteins: inositol-requiring kinase 1α (IRE1α), double-stranded RNA-dependent protein kinase-like ER kinase (PERK) and activating transcription factor-6α (ATF6α). Under normal conditions, these three sensors are maintained in an inactive state by binding to the ER chaperone protein glucose-regulated protein 78 (GRP78). Upon ER stress, GRP78 is released, allowing activation of PERK, IRE1α, and ATF6, and their downstream signaling pathways. Liver samples from NAFLD patients demonstrated the activation of the UPR, although there was a variable degree of UPR activation depending on the stages of the disease [50, 52]. Moreover, after caloric restriction, obese subjects showed weight loss together with ameliorated IR, reduced ER stress markers and improved mitochondrial function [53], suggesting the link between ER stress and abnormal lipid metabolism/IR.

**Fig. 3:**
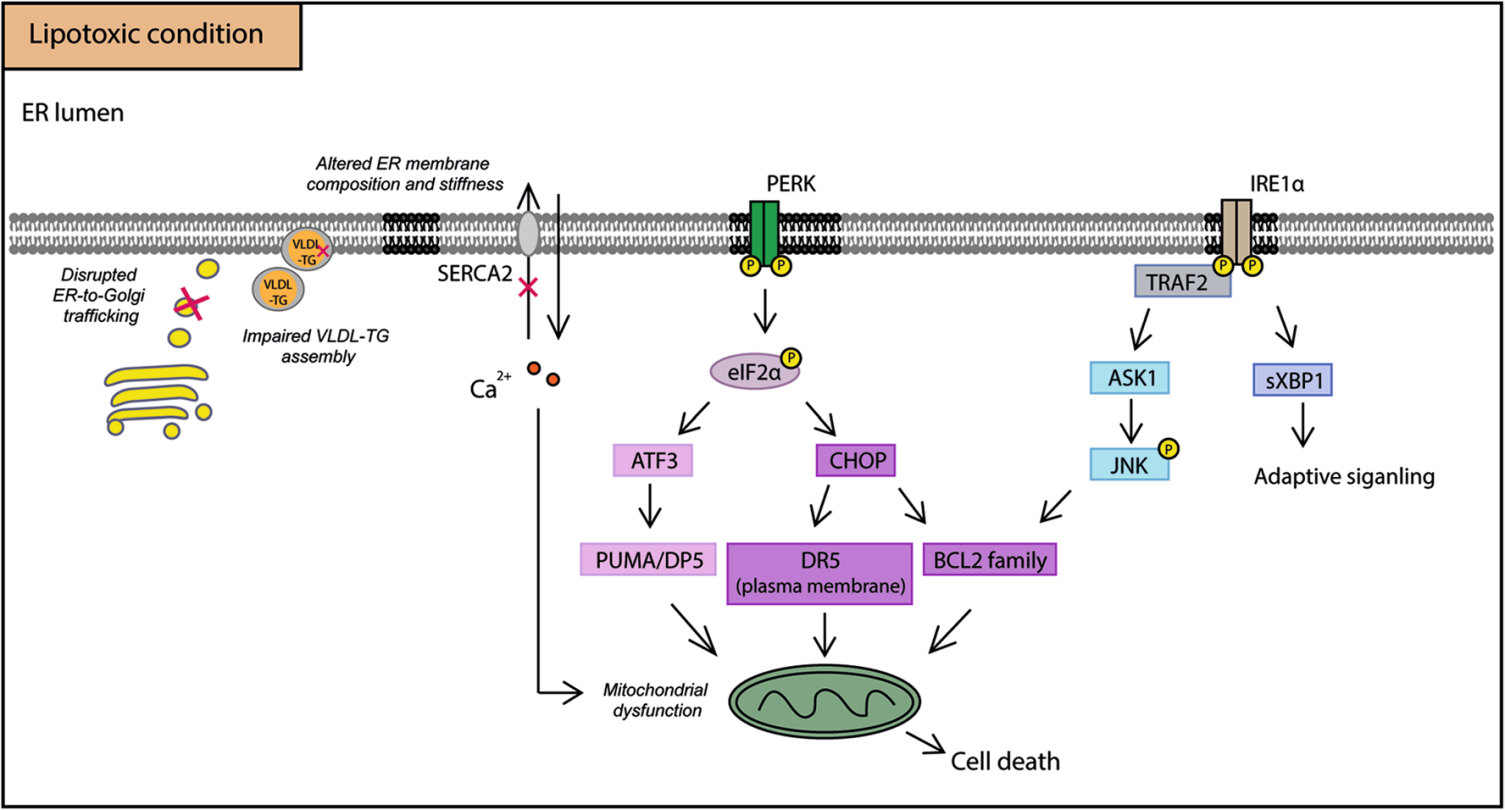
Lipotoxic UPR induces hepatic cell death. Under lipotoxic condition, several factors are implicated in the occurrence of ER stress, including disrupted ER-to-Golgi protein trafficking, altered ER membrane composition and stiffness, impaired VLDL-TG assembly and the change of Ca^2+^ gradient by the decreased level of SERCA2 activity. PERK is activated and leads to the phosphorylation of eIF2α, subsequently causes the activation of downstream ATF3- and CHOP-related apoptotic signaling pathways. IRE1α could also be activated by toxic lipids, thus stimulates TRAF2/ASK1/JNK signaling pathway. On the other hand, spliced XBP1 mediates adaptive UPR, which aims to alleviate ER stress. Together with apoptotic UPR, the massive efflux of Ca^2+^ from ER could result in mitochondrial dysfunction and the initiation of apoptotic cell death. *VLDL* very-low-density lipoprotein, *SERCA2* sarco/endoplasmic reticulum Ca^2+^-ATPase 2, *ATF3* activating transcription factor 3, *ASK1* apoptosis signal-regulating kinase 1, *CHOP* C/EBP homologous protein, *DR5* death receptor 5, *DP5* death protein 5, *eIF2α* eukaryotic initiation factor 2α, *TRAF2* TNF receptor-associated factor 2, *IRE1α* inositol-requiring kinase 1α, *JNK* c-Jun N-terminal kinase, *PERK* double-stranded RNA-dependent protein kinase-like ER kinase, *PUMA* p53 upregulated modulator of apoptosis, *sXBP1* spliced X-Box Binding Protein 1

Both in vivo and in vitro studies indicated that lipotoxicity is an important driver of ER stress in the context of NAFLD. Animals fed a diet enriched in saturated fat exhibited increased ER stress markers (e.g. X-box binding protein 1 (sXBP1) and GRP78) and liver injury, whereas diets with high unsaturated fat or starch did not induce ER stress or liver injury [30, 54]. In vitro studies demonstrated that hepatocytes challenged with several kinds of saturated fatty acids or lysophosphatidylcholine (LPC) showed activation of the apoptotic UPR and subsequent cell death [55, 56] (Fig. 3). Mechanistically, the activation of apoptotic UPR by toxic lipids has been linked to several pro-apoptotic proteins. For example, C/EBP homologous protein (CHOP) is one of the best-known pro-apoptotic proteins in response to ER stress. It regulates the expression of downstream BCL2 family proteins, causes Ca^2+^ release from ER and increases the expression of death receptor 5 (DR5) [50]. Meanwhile, the PERK/ATF3 pathway was shown to mediate palmitate-induced induction of the BH-3 only protein p53 upregulated modulator of apoptosis (PUMA) and death protein 5 (DP5) [57]. Besides, IRE1 was demonstrated to orchestrate cell fate by controlling XBP1-mediated adaptive signaling and stimulating TRAF2/ASK1, subsequently activating JNK and p38 MAPK that promote apoptosis [50, 58]. In addition, the release of Ca^2+^ from the ER to the cytosol is another important player involved in the intrinsic (mitochondrial) pathway of apoptotic cell death [50, 59].

Apart from regulating apoptotic cell death, the UPR also plays an important role in mediating lipid metabolism and IR. Among the UPR proteins, XBP1 is one of the most important regulators of lipogenesis and VLDL secretion, via directly and indirectly controlling the expression of critical genes involved in fatty acid synthesis [60]. PERK/ATF4 and ATF6 are also involved in hepatic steatosis. Genetic ablation of ATF4 resulted in hepatic steatosis in response to chemical induction of ER stress [61]. The nuclear form of ATF6α inhibits the transcriptional activity of sterol regulatory element-binding protein 2 (SREBP2) and blocking ATF6 inhibited hepatic mitochondrial fatty acid oxidation by decreasing the transcriptional activity of PPARα [62]. Under lipotoxic conditions, these regulatory pathways were disturbed. Lipotoxic ER stress also contributes to IR, which was mechanistically associated with the lipotoxic inhibition of IRE1α and its downstream transcription factor XBP1 via Bax inhibitor-1 (BI-1), an ER membrane protein [63]. Moreover, attenuation of palmitate-induced ER stress reduced IR and reversed the changes in insulin signaling pathways [64].

### Mitochondrial dysfunction

As discussed previously in 2.3, even though the increased fatty acid flux and fatty acid uptake in the liver did not lead to clear alterations in mitochondrial β-oxidation, there is a consistent observation of mitochondrial dysfunction in NASH. Liver biopsies obtained from both NASH patients and rodent NAFLD models demonstrated defective mitochondrial respiratory chain function together with altered mitochondrial structure, presented as enlarged mitochondria, rounded cristae and loss of the typical dense mitochondrial granules, as well as an epigenetic modification on mitochondrial DNA [65, 66]. Compelling evidence shows that fatty acids (e.g. SFAs and ceramide) exhibit detrimental effects on mitochondrial function. Treatment with palmitic acid leads to a doubling in oxygen uptake rate at early stage and overproduction of reactive oxygen species (ROS), which causes oxidative modification of proteins and lipids and/or disrupts signaling pathways via inactivation of phosphatases [67]. It also induces the release of mitochondrial DNA from cells [68]. Moreover, palmitic acid-induced activation of JNK is related to the mitochondrial protein Sab (Sh3bp5) [69]. FFAs also indirectly cause mitochondrial injury by inducing lysosomal permeabilization, which causes activation of pro-apoptotic members of the Bcl-2 family that subsequently induce mitochondrial permeabilization and apoptotic cell death [70, 71].

### Lysosomal dysfunction and impaired autophagy

Lysosomal dysfunction is described as reduced lysosomal acid lipase (LAL) activity, disturbed lysosomal acidification and lysosomal permeabilization and has been reported in NAFLD. LAL, hydrolyzing cholesteryl esters and TGs in lysosomes, play a role in lipid metabolism as well as in cell death in a caspase-dependent or -independent way. Clinical studies have shown that both the hepatic and serum LAL activity are reduced in NAFLD patients [72, 73]. Another study reported that in pediatric patients with NAFLD, the reduced serum LAL activity correlates with the severity of NAFLD-induced liver fibrosis but not with NAFLD [74], indicating its association with liver injury. Moreover, a case study highlighted the importance of considering LAL in the differential diagnosis of NAFLD, as its deficiency may cause a failure of treatment even when the patient achieves weight reduction [75].

Lysosomal acidification is disturbed in NAFLD patients, as demonstrated by the decreased number of acidic organelles by acridine orange staining and reduced level of mature cathepsin D [76]. Interestingly, lysosomal acidity has been shown to be mainly affected by exogenous but not endogenous fatty acids [77]. Exogenous fatty acids induce the redistribution of cathepsin B into the cytoplasm via promoting the translocation of Bax to lysosomes [71, 78]. Moreover, FFA-induced lysosomal permeabilization could lead to mitochondrial dysfunction since both pharmaceutical and genetic inhibition of cathepsin B preserved mitochondrial function, demonstrating the role of lysosomes in the initiation of the intrinsic pathway of apoptosis [70, 71].

Moreover, autophagy is impaired in NASH, which is associated with defective autophagosome formation and reduced lysosomal acidification [76]. Toxic fatty acids inhibit autophagic flux, which can lead to reduced mitophagy, reduced lipophagy, concomitant ER stress and oxidative stress, aggravating subcellular dysfunction [79]. On the other hand, these subcellular responses also affect autophagy, e.g. the activated UPR impairs selective autophagy by inhibiting autophagosome–lysosome fusion [80]. Promoting autophagy is considered as a therapeutic strategy in the treatment of NAFLD, however, a cell-specific outcome needs to be noted [79, 81].

### JNK activation

As described above, JNK, a classic molecular regulator of cellular injury, can be activated by mitochondrial proteins, UPR signaling and ROS production in NASH. The accumulation of toxic lipids may cause sustained activation of JNK via ROS and MAP3K activation, while inhibition of JNK attenuates FFA-induced lipoapoptosis [69]. Noteworthy, the detrimental effects of JNK are mostly attributed to the JNK1 isoform. Investigations on the role of the JNK2 isoform suggest that JNK2 could be protective against cell injury [82, 83]. Evidence also exists for the central role of JNK in obesity and IR. The absence of JNK1 results in reduced obesity and improved IR [83]. These effects may be associated with the increased expression of PPARα target genes [69].

### Mode of cell death involved in the pathogenesis of NAFLD

**Fig. 4:**
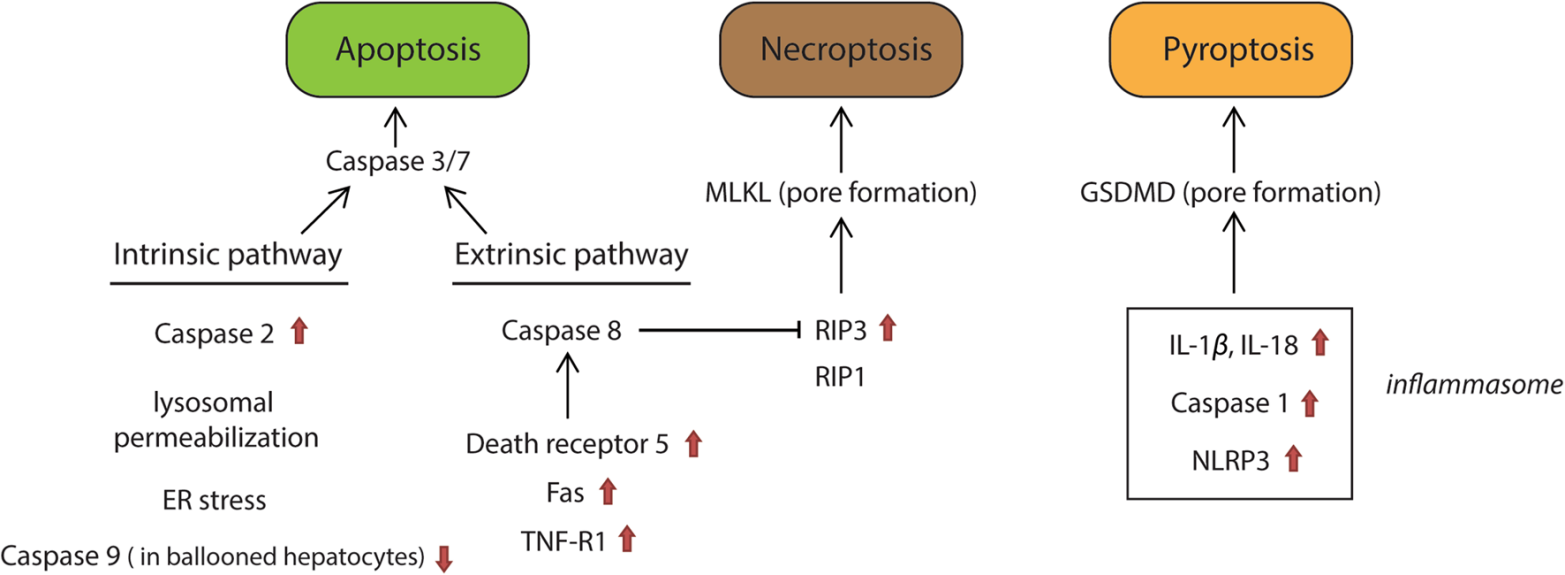
Modes of cell death in NASH. Apoptosis, necroptosis and pyroptosis have all been described in NASH. Apoptosis, which can be demonstrated by caspase 3/7 activation, is triggered through both intrinsic and extrinsic pathways in NASH. Lysosomal dysfunction and ER stress are involved in the initiation of intrinsic pathway. In addition, caspase 2 is increased in NASH, whereas caspase 9 level is reduced in ballooned hepatocytes. In the extrinsic pathway, death receptor 5, apoptosis antigen 1 (Fas) and tumor necrosis factor receptor 1 (TNF-R1) are increased in NASH leading to activation of caspase 8 and induction of apoptosis. Necroptosis is characterized as a mixed lineage kinase domain-like pseudokinase (MLKL) related pore formation in the plasma membrane. It is induced by Receptor-Interacting Protein 3 (RIP3) and RIP1. Caspase 8 inhibits RIP3 activation. Pyroptosis is characterized as gasdermin D (GSDMD) mediated pore formation in the plasma membrane. Its activation is accompanied by inflammasome formation, which involves the maturation of caspase 1, IL-1β and NLRP3. Arrows (red) indicate the up- or down-expressions of specific proteins in NAFLD. *IL-1β* interleukin-1β, *NLRP3* NACHT, LRR and PYD domains-containing protein 3

Hepatic cell death is a common characteristic and an important driver in the progression of NASH, as well as the end-point outcome of lipotoxicity (Fig. 4). Several modes of cell death (apoptosis, necroptosis and pyroptosis) have been described in NASH and linked to lipotoxicity-induced cell death. Evidence for all modes of cell death have been presented, but it is still a matter of debate which form of cell death is predominant in the context of NAFLD. The evidence for apoptotic cell death is based on positive TUNEL staining and immunohistochemistry for (cleaved) caspase 3/7 in liver biopsies as well as increased plasma levels of the cytokeratin-18 fragment and soluble Fas from NASH patients (but not from patients with simple steatosis). Lipotoxicity resulting in apoptosis has been termed lipoapoptosis and involves both the intrinsic or mitochondrial/lysosomal pathway as well as the extrinsic or death receptor pathway. The activation of the intrinsic apoptotic pathway is closely associated with the above mentioned subcellular dysfunctions (ER stress, mitochondrial dysfunction, lysosomal dysfunction and JNK activation) and includes the activation of caspase 2, an initiator caspase. Caspase-2 was markedly upregulated in injured hepatocytes in NASH patients and knockout of caspase 2 ameliorated steatohepatitis in mice [84, 85]. Furthermore, lipoapoptosis also involves the activation of caspase 2 [86]. In contrast, the expression of caspase-9 was shown to be reduced in ballooned hepatocytes in NASH specimens, which was explained by increased hedgehog signaling to prevent ballooned hepatocytes from death [87]. On the other hand, several extrinsic apoptotic receptors (death receptor 5, Fas receptor and TNF-R1) are significantly increased in liver samples from patients and animal models of NASH [88]. Steatotic hepatocytes demonstrated increased sensitivity to TRAIL- or Fas-mediated cell death. Genetic deletion of TRAIL receptor suppressed steatohepatitis and hepatic apoptosis [88, 89].

Several recent studies emphasized the role of necroptosis in the pathogenesis of NASH [90–92]. Both the regulatory factor RIP3 and the execution factor MLKL of necroptosis are expressed at low levels in the normal liver [93], NASH patients and dietary NASH mouse models demonstrated increased expression of RIP3 in the liver and elevated levels of RIP1 and MLKL in the serum [94, 95]. Moreover, inhibition of RIP1 by a specific RIP1 inhibitor protected against HFD-induced hepatic steatosis, inflammation and fibrosis in mice [95]. Deletion of RIP3 induced a shift from necroptosis to apoptosis in a HFD-mouse model, correlating with increased inflammation and hepatocyte apoptosis, as well as an early fibrotic response. In our studies, we observed that palmitic acid alone induced necrosis rather than apoptosis in primary rat hepatocytes [96], supporting a major role for necroptotic cell death. Moreover, inhibition of RIP1 with necrostatin-1 or siRNA knockdown of RIP3 reduced palmitic acid-induced cytotoxicity in vitro [97]. Therefore, lipotoxicity may not directly induce lipoapoptosis in primary hepatocytes.

Pyroptosis has also been implicated in the pathogenesis of NASH. Pyroptosis involves the activation of caspase-1 by the inflammasome multi-protein complex resulting in the activation of several pro-inflammatory cytokines and the cleavage of gasdermin proteins, which mediate pore formation in the plasma membrane and ultimately result in cell death. Liver samples of NASH patients and rodent models of NASH showed increased expression of caspase 1, gasdermin D (GSDMD) and inflammasome components compared to the simple steatosis stage [98, 99]. Saturated fatty acids as well as ceramide, but not unsaturated fatty acids, were shown to increase the level of inflammasome components in several types of hematopoietic cells. Palmitic acid also increases mRNA expression of NLRP3 in hepatocytes and triggers the release of danger signals, which in turn activate the inflammasome and the release of IL-1β and TNFα from liver mononuclear cells [100]. These effects may be associated with impaired mitophagy [101]. Furthermore, palmitic acid and LPS showed synergy in activating caspase 1 in hepatocytes [102]. In addition, it was proposed that pyroptosis might not only play a role in chronic inflammation but also in hepatic lipogenesis and fibrogenesis. Inhibition of caspase 1 or GSDMD ameliorated hepatic steatosis, inflammation and fibrosis in experimental NAFLD models [98]. In summary, available literature indicates that several modes of cell death (apoptosis, necroptosis, pyroptosis) co-exist in the context of NAFLD. It is not possible to identify a dominant mode of cell death. The ‘mixed phenotype’ of cell death in the context of NASH has major implications for the selection of therapeutic targets.

### Extracellular vesicles (EVs)

Extracellular vesicles (EVs) are nano-sized vesicles that can be categorized into three subgroups: exosomes, microvesicles and apoptotic bodies. As an important mode of intercellular communication, EVs play a critical role in the pathogenesis of NAFLD and its actions in NAFLD were reviewed recently [103]. With regard to the increased secretion of EVs during the progression of NAFLD, experimental studies demonstrated that toxic lipids significantly increase the production of hepatocyte-derived EVs, which act as mediators of paracrine signaling, causing hepatic stellate cell activation, angiogenesis and activation of macrophages leading to inflammation and chemotaxis [103–105]. Mechanistically, the detrimental effects of EVs derived from toxic lipid-treated hepatocytes were attributed to miRNAs, proteins and lipids transported by EVs, e.g., miR-128-3p, inflammasome components, and C–X–C motif ligand 10 (CXCL10) [103].

The production of EVs is a regulated process. Palmitic acid-induced release of EVs was associated with the activation of the IRE1α/XBP-1 signaling pathway [106]. Meanwhile, the release of CXCL10-bearing EVs from hepatocytes was induced by mixed-lineage protein kinase 3 (MLK3) activation [107]. In addition, the inhibition of rho-associated protein kinase 1 (ROCK1) reduced the release of hepatocyte-derived EVs [104].

### Hypoxia

Both clinical and experimental evidence show that the development of NAFLD is closely associated with hypoxia, which could be caused by e.g. obstructive sleep apnea syndrome (OSAS) affecting 35–45% of obese individuals [108]. OSAS/hypoxia acts as a trigger for localized hepatic oxidative stress and inflammation that promotes the progression of NASH and hepatic fibrosis [109, 110]. Our recent studies revealed that hypoxia is an important factor that aggravates the toxic effects of lipids in hepatocytes. Hypoxic conditions elevated the inflammation in steatotic hepatocytes and increased the release of EVs derived from fat-laden hepatocytes, which stimulate the activation of hepatic stellate cells and the inflammatory phenotype in Kupffer cells [111, 112]. Vice versa, simple steatosis enhances the sensitivity of hepatocytes to hypoxic injury [113], though more detailed studies are needed. At the molecular level, the hypoxia signaling pathways are transcriptionally regulated by hypoxia-inducible factors (HIFs), which consist of three isoforms: HIF-1, HIF-2 and HIF-3. Both HIF-1α and HIF-2α have been shown to be associated with hepatic lipid metabolism, liver injury and the development of liver fibrosis [114, 115]. Therefore, hypoxia acts as a factor that worsens (or is exacerbated by) lipotoxicity and, importantly, it could be a link between simple steatosis and NASH.

## Concluding remarks

The excessive hepatic lipid flux, which is derived from the disturbed metabolic balance of lipids, changes the absolute and relative levels of the various lipid species in the liver. Several lines of evidence highlight the critical role of non-TG lipids, such as SFAs, free cholesterol and ceramides, in the progression to NASH by triggering inflammation, cellular damage and fibrogenesis. At the subcellular level, the detrimental actions of toxic lipids cause the dysfunction of several cellular compartments as discussed in Sect. 3. Moreover, the interactions between these compartments aggravate lipotoxicity. Additionally, some “independent” factors, such as hypoxia, can further aggravate lipotoxic effects.

Therefore, counteracting lipotoxicity has emerged as a therapeutic strategy for NASH. As discussed in this review, TG is believed to be a non-toxic lipid. Promoting TG synthesis or lipid droplet biogenesis (e.g. by monounsaturated fatty acids) has been shown to counteract FFA-induced cellular toxicity, which is possibly due to the incorporation of toxic fatty acids into inert TGs [30]. Moreover, reducing oxidative stress (e.g. by vitamin E or metformin) also alleviates lipotoxicity [96, 116]. Furthermore, the use of chemical chaperones [e.g. tauroursodeoxycholic acid (TUDCA)] or stimulating the expression of ER chaperones like GRP78 (e.g. by hesperetin) to relieve ER stress also inhibit lipotoxicity [117, 118]. Candidate drugs that are currently in clinical trials are mainly aimed at reducing the lipid accumulation in the liver [3, 119]. However, investigating the consequences of changes in the composition of lipids in NAFLD might also be an important strategy to identify novel targets for intervention. In conclusion, future research on the relative contribution of lipotoxicity in the pathogenesis of NAFLD and the elucidation of the effects of various lipid species are necessary and will provide directions for drug design as well as targeted therapies for NAFLD.

## Abbreviations

AA: Arachidonic acid
ATF6: Activating transcription factor-6
CHOP: C/EBP homologous protein
CIH: Chronic intermittent hypoxia
CVD: Cardiovascular disease
CYPs: Cytochromes P450
DG: Diacylglycerol
DGATs: Diacylglycerol acyltransferases
DHA: Docosahexaenoic acid
DNL: De novo lipogenesis
DPA: Docosapentaenoic acid
EPA: Eicosapentaenoic acid
ER: Endoplasmic reticulum
FAO: Fatty acid oxidation
FAT/CD36: Fatty acid translocase
FATPs: Fatty acid transport proteins
FFAs: Free fatty acids
GPL: Glycerophospholipid
GRP78: Glucose-regulated protein 78/immunoglobulin-heavy-chain-binding protein
GSDMD: Gasdermin D
HIFs: Hypoxia-inducible factors
IR: Insulin resistance
IRE1α: Inositol-requiring kinase 1α
JNKs: c-Jun N-terminal kinases
LAL: Lysosomal acid lipase
LDL: Low-density lipoprotein
LPL: Lipoprotein lipase
LPS: Lipopolysaccharides
LyPC: Lysophosphatidylcholine
MAFLD: Metabolic associated fatty liver disease
MLKL: Mixed lineage kinase domain-like
NAFL: Non-alcoholic fatty liver
NAFLD: Non-alcoholic fatty liver disease
NASH: Non-alcoholic steatohepatitis
NLRP3: NACHT, LRR and PYD domains-containing protein 3
OSAS: Obstructive sleep apnea syndrome
PC: Phosphatidylcholine
PE: Phosphatidylethanolamine
PERK: Double-stranded RNA-dependent protein kinase-like ER kinase
PI: Phosphatidylinositol
PUMA: p53 upregulated modulator of apoptosis
RIP: Receptor-interacting protein
ROS: Reactive oxygen species
SREBPs: Sterol regulatory element binding proteins
SERCA2: Sarco/endoplasmic reticulum Ca^2+^-ATPase 2
TGs: Triglycerides
TRAIL: TNF-related apoptosis-inducing ligand
UPR: Unfolded protein response
VLDL: Very-low-density-lipoprotein
WAT: White adipose tissue
